# The Impact of the Coronavirus Disease 2019 (COVID-19) Pandemic on Postgraduate Training in Obstetrics and Gynaecology: A Global Perspective

**DOI:** 10.7759/cureus.83783

**Published:** 2025-05-09

**Authors:** Kavita Schapira, Abha Govind, Nisha Lakhi

**Affiliations:** 1 Obstetrics and Gynaecology, Richmond University Medical Center, Staten Island, USA; 2 Obstetrics and Gynaecology, North Middlesex University Hospital NHS Trust, London, GBR

**Keywords:** covid-19, medical resident education, obstetrics and gynaecology, postgraduate medical education (pgme), world pandemic

## Abstract

This paper reviews survey studies to assess the global impact of the coronavirus disease 2019 (COVID-19) pandemic on obstetrics and gynaecology (O&G) training from the perspectives of trainees and programme directors (PDs). An electronic literature review of the PubMed database was conducted from March 2020 to December 2024 for topics pertaining to the COVID-19 pandemic and trainee education to identify pertinent validated and non-validated survey studies. Ten studies were identified that analysed the impact of the COVID-19 pandemic on postgraduate training in O&G. All 10 studies utilised non-validated questionnaires. Two studies (total respondents: N=182) assessed the viewpoint of O&G PDs, and eight studies (total respondents: N=1416) solicited the perspectives of postgraduate trainees (PGTs) in O&G. Countries represented include Brazil, the European Union (EU) (encompassing 25 different countries), Germany, India, Italy, Sweden, Turkey, the United Kingdom, and the United States. The themes identified were as follows: (1) trainee reassignment, (2) restructuring of didactic and research activities, and (3) loss of clinical training opportunities within the specialty and its impact on trainees' mental health. Understanding this impact is key to enable better planning in similar pandemics in the future. The review suggests that there were both positive and negative impacts of the pandemic that may forever shape trainee education.

## Introduction and background

The coronavirus disease 2019 (COVID-19) pandemic affected residency training in obstetrics and gynaecology (O&G) globally. Although there is variation in the structure and format of residency training throughout the world, the challenges resulting from the pandemic have been similar [1,2]. Residents are expected to achieve surgical, clinical, and educational milestones as they progress through training. O&G at its core is a surgical specialty. While some clinic visits can be conducted virtually, a large part of this specialty's nature is dependent on face-to-face interaction and instruction. Daily practice, with sufficient theoretical preparation, is critical in gaining autonomy in carrying out clinical duties and for the overall development of surgical skills [3,4]. While obstetric care continued, the redeployment of postgraduate trainees (PGTs) to designated COVID-19 wards and postponement of elective benign gynaecology surgery led to a decrease in operating volumes. This may have had a significant impact on surgical progression.

This review surveys studies to assess the global impact of this pandemic on O&G training from the perspectives of trainees and programme directors (PDs). We highlight common themes across several O&G programmes and discuss how the pandemic changed PGTs. Understanding this impact is important to ensure trainees are competent and well-supported should future pandemics arise. 

## Review

**Figure 1:**
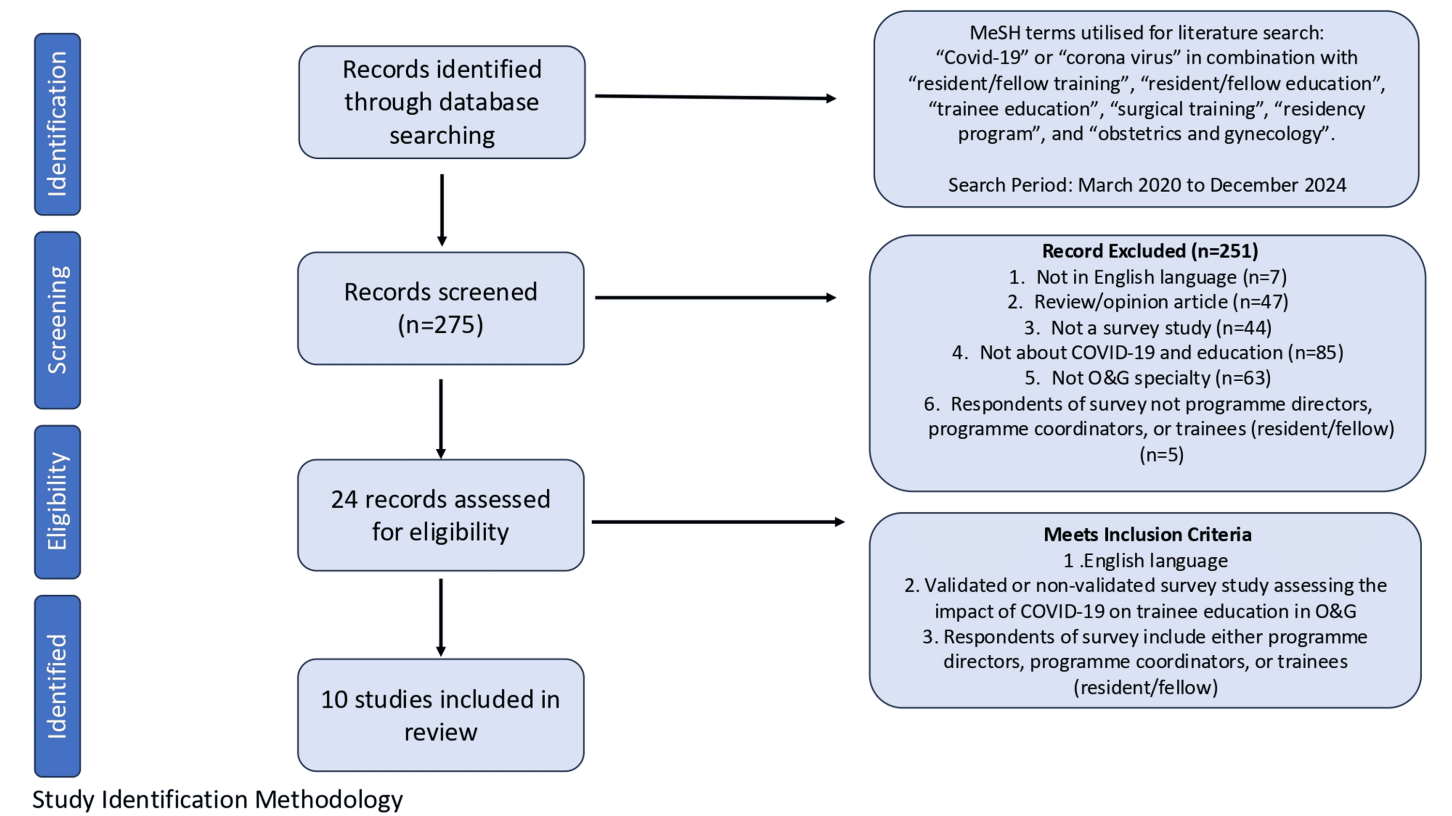
Study selection diagram O&G: obstetrics and gynaecology; MeSH: Medical Subject Headings

An electronic literature review in the PubMed database was conducted from March 2020 to December 2024 for topics pertaining to the COVID-19 pandemic and its effect on trainee education in O&G. The literature search used the terms "Covid-19" or "corona virus" in combination with "postgraduate/resident/fellow training", "resident/fellow education", "trainee education", "surgical training", "residency programme", and "Obstetrics and Gynaecology". Studies were reviewed for inclusion based on subject matter pertinent to trainee education in O&G in relation to the COVID-19 pandemic. English language papers were considered eligible if they were published in a peer-reviewed journal and included either validated or non-validated questionnaires assessing the impact of COVID-19 on O&G postgraduate/resident/trainee education (Figure 1). Bias from each of these studies was assessed across three different domains: tool validation, response rate, and selective reporting. These three domains were then used to assess the overall bias risk of the individual studies (Table 1). Quantitative synthesis was not performed on the extracted data given that the purpose of this analysis was narrative in nature. 

**Table 1:** Risk of bias assessment

References	Validation of tools	Response rate	Selective reporting	Overall bias assessment
Bitonti et al. [1]	Moderate	Moderate	Moderate	Moderate
Kraus et al. [2]	Moderate	Moderate	High	High
Boekhorst et al. [3]	Moderate	Moderate	High	High
Butler et al. [4]	Low	Moderate	High	Moderate
Brito et al. [5]	Moderate	High	High	High
Riedel et al. [6]	Moderate	Low	Moderate	Moderate
Gothwal et al. [7]	High	Moderate	Moderate	High
Wådell et al. [8]	High	Moderate	Moderate	High
Topçu et al. [9]	High	Moderate	High	High
Mallick et al. [10]	Moderate	Moderate	Moderate	Moderate

For this paper, residents and fellows are described as PGTs. Data points extrapolated from each study included source, country, study type, target respondents of the survey, and response rate. Results from the included studies are presented using thematic analysis along with pertinent key findings. Themes were identified after a review of each paper. The three authors discussed the themes and decided on the final selection by consensus. Registration of subjects or formal institutional approval was not applicable to this literature review study. 

Ten articles were identified from the literature search that analysed the impact of COVID-19 on PGT in O&G using surveys (Table 2). All 10 studies utilised non-validated questionnaires that were distributed using internet or e-mail resources. Of the 10 studies, two (total respondents: N=182) [5,6] assessed the viewpoint of O&G PDs, and eight studies (total respondents: N=1416) [1-4,7-10] solicited the perspectives of PGTs in O&G. Studies were from Brazil (N=1), the European Union (EU) encompassing multiple countries (N=1), Germany (N=1), India (N=1), Italy (N=1), Sweden (N=1), Turkey (N=1), the United States (N=2), and the United Kingdom (N=1). The inclusion and exclusion criteria were defined in Figure 1. The themes identified were as follows: (1) trainee reassignment, (2) restructuring of didactic and research activities, and (3) loss of clinical training opportunities within the specialty and its impact on trainees' mental health. 

**Table 2:** Included studies O&G: obstetrics and gynaecology; COVID-19: coronavirus disease 2019; KSS: Kent, Surrey, and Sussex; COPE Staff: COVID-19 in Pregnancy and Early Childhood Staff

Author	Country/organisation	Questionnaire type	Population sample
Bitonti et al. [1]	Italy	Non-validated cross-sectional survey	Italian O&G resident trainees (N=476); response rate: 51%
Kraus et al. [2]	United States/American College of Obstetricians and Gynecologists (ACOG)	Non-validated cross-sectional survey consisting of 28 questions	US O&G resident trainees (N=95); response rate: not reported
Boekhorst et al. [3]	European Union/European Network of Trainees in Obstetrics and Gynaecology (ENTOG)	Non-validated online cross-sectional survey posted on the ENTOG social media site consisting of 40 questions	European trainee in O&G at the time of the COVID-19 pandemic (N=125); response rate: not reported
Butler et al. [4]	United States/American Urogynecologic Society (AUGS)	Non-validated online survey consisting of 24 questions	US fellowship trainees in female pelvic medicine and reconstructive surgery (N=88); response rate: 52%
Brito et al. [5]	Brazil/Brazilian Federation for Gynecologists and Obstetrics (FEBRASGO)	Non-validated email questions sent to all accredited O&G postgraduate medical residency programmes	Brazilian O&G programme directors (N=152); response rate: 30.4%
Riedel et al. [6]	Germany/Young Forum of the German Society for Gynecology and Obstetrics	Non-validated email questionnaire consisting of 42 items	Teaching coordinators of O&G departments at public and private German university hospitals (N=30); response rate: 73.2%
Gothwal et al. [7]	India	Non-validated cross-sectional questionnaire-based online survey consisting of five sections: demographics, information regarding COVID-19 status, clinical workload, teaching and research, and psychological impact	Indian O&G postgraduate trainees (N=280); response rate: not reported
Wådell et al. [8]	Sweden/Swedish Society of Obstetrics and Gynecology (SFOG)	Non-validated email survey consisting of 28 questions distributed via the COPE Staff cohort study	Swedish O&G resident trainees (N= 162); response rate: not reported
Topçu et al. [9]	Turkey/European Network of Trainees in Obstetrics and Gynaecology (ENTOG)	Non-validated cross-sectional survey consisting of 40 questions	Turkish O&G resident trainees (N=103); response rate: not reported
Mallick et al. [10]	United Kingdom	A 33-question non-validated email survey was sent to all O&G trainees in KSS by mail	British O&G trainees based in KSS (N=87); response rate: 69%

Trainee reassignment

Due to COVID-19, O&G PGTs were redeployed to services outside of their specialty. Seven [2-5,7-9] of the 10 studies assessed trainee reassignments. Six studies reported rates of their PGTs being deployed to COVID-19-specific wards, ranging from 13% to 86.6% across countries, with the lowest rate being reported by the United Kingdom [1] and the highest rates in India [7]. In many countries, PGTs felt inadequately prepared to take care of COVID-19 patients. Thirty-three percent of respondents in the Brazilian study [5] stated that they did not get appropriate training for COVID-19 cases, while 46.5% of PGTs in Turkey [9] were reassigned to the COVID-19 intensive care unit without any training. In the United Kingdom, although 75% of respondents had access to personal protective equipment (PPE), only 56% of them felt that they were well-trained in how to use this [10]. Twenty percent of respondents from a US study reported they did not have access to adequate PPE, and 47% reported violating the 80 hours per week duty requirement. Sixteen percent had less than four days off per month on average during the pandemic [2].

Despite being largely relocated to wards outside their specialty, 80.6% of PGTs in India felt they got adequate PPE training, and 93.9% felt appropriately trained on COVID-19-related care [7]. Only 15% of PGTs in the EU survey reported being deployed to work on COVID-19-specific units [3]. In the Turkish study, 65.3% of trainees reported being sent to COVID-19 units [9]. In Sweden, 27% of O&G trainees reported being transferred to other healthcare institutions, and 30% worked in a COVID-19-specific unit [8]. They further noted that 12% of the trainees performed clinical assignments normally performed by other professions such as a nurse, midwife, or assistant nurse [8]. In Brazil, 34% of the surveyed PDs reported that their PGTs were relocated to activities not related to O&G [5]. Additionally, 20% of the PDs responded that their institution relocated their O&G PGTs to assist at intensive care units specifically designated for COVID-19 patients [5]. In a US study, 43% of urogynecology PGTs reported being redeployed, but not sent to COVID-19-specific wards [4]. Most (85%) of the redeployments were within the O&G department itself, consisting of general O&G responsibilities [4]. 

Three studies reported increased rates of working from home [3,4,8]. In the EU, working from home was introduced to many trainees. It was reported that 38% of the PGTs spent at least some part of their working hours from home and 19% of those reported 20 hours or more of work from home per week [3]. In Sweden, 88% of the PGTs reported working from home during the pandemic, while 69% reported working extra hours overall [8]. The study from the United States reported a reduction in working hours for urogynecology trainees. Only 55.6% of respondents were working on-site. Of these trainees, 42% reported working only 1-10 hours per week and 20% working 10-20 hours per week [4].

Restructuring of didactic and research activities

Prior to the onset of the COVID-19 pandemic, many programmes utilised in-person didactic sessions as their main source of PGT. However, with the social distancing requirements implemented, programmes reported that they had to reassess both their curriculums and method of dissemination. Four [5-8] of the 10 studies discussed the restructuring of didactic education in response to the pandemic. In the US study, 74% had an interruption of regular schedule [2]. In India, a total suspension of didactic education was reported by 27.9% of PGTs [7]. In Sweden, a majority of PGTs (94%) stated that the continuity of their education programme had been affected by the pandemic [8]. 

Three studies discussed the implementation of virtual learning [5-7]. In India, 54.7% of respondents reported the adoption of an online model of training at their institution [7]. However, the study acknowledged the limitations of this method (e.g., connection issues, limited interactions, and an increase in distractions) [7]. In Brazil, 85% of PDs reported that a virtual online course was created for their programme; however, almost none (95%) included a home surgical skill training [5]. Additionally, 15% of the PDs did not have access to a virtual supportive environment (e.g., internet connection or access to Zoom/Google Meet) to maintain classes [5]. In contrast, most of the teaching coordinators (58%) surveyed in the German study reported that the implementation of the online modules had been swift and uncomplicated and their universities provided adequate technical support (58%) [6]. The study also found that many (45%) of the teachers for the O&G programme felt that the virtual didactic curriculum was an adequate replacement for the in-person lectures [6]. 

Two studies reported loss of research opportunities secondary to the pandemic [7,8]. In Sweden, access to research experiences was reported by only 25% of PGTs. Of these, one-fourth reported a reduction in allocated research time due to the pandemic [8]. In India, 74.3% of PGTs reported that their academic thesis goals were not achieved due to the pandemic [7]. One study from the United States reported increased research opportunities due to trainees working from home. A majority of respondents (37%) spent 11-15 hours per week on research, and 20% reported 16-20 hours per week [4].

Loss of clinical training opportunities within the specialty and its impact on trainees' mental health

The specialty of O&G is heavily dependent on in-person training opportunities. However, during the COVID-19 pandemic, many elective surgical cases and other clinical learning experiences were put on indefinite hold. Nine of the 10 studies reported loss of clinical training opportunities that were a result of COVID-19 [1-5,7-10]. In the United Kingdom, 43% of trainees believed that the pandemic had negatively affected their obstetric training experience compared to almost 99% who felt their benign gynaecology surgical training experience had been negatively affected [10]. In Turkey, a majority of the PGTs (98%) reported many routine surgeries were decreased or were cancelled. In addition, 63% reported that their surgical skills were hindered by the reduced number of surgeries [9]. The Swedish study reported that more than two-thirds of the respondents (70%) had performed fewer surgeries than before the pandemic [8]. In the EU study, 67% of respondents reported a reduction in surgical opportunities due to the cancellation of procedures, and only 5% stated that they met the goals for their surgical competencies [3]. PDs in Brazil reported that gynaecological surgeries were the most affected, as noted by 72% of respondents [5]. A study of urogynecology PGTs from the United States noted that only 31% were performing specialty-specific surgery during the pandemic [4]. Another US study reported that over 80% of fourth-year PGTs said their gynaecological training had suffered with 70% expressing a lack of confidence in their ability to independently practice gynaecological surgery after graduating [2].

Four studies [3,5,9,10] reported loss of training opportunities in the outpatient setting. In the United Kingdom, 46% and 93% of the PGTs reported that their respective antenatal clinic and gynaecology clinic experiences had been negatively affected [10]. In Turkey, 67% of the PGTs reported insufficient outpatient clinic experience to meet education targets [9]. In Brazil, 23% of PDs reported cancelled gynaecological outpatient clinics [5]. In the EU study, outpatient family planning was one of the most affected services as 34% of PGTs reported the procedures were cancelled [3]. 

Four studies [1,7,8,10] noted the negative impact that the loss of clinical opportunities had on the quality of training. In Sweden, 69% of the respondents were worried that the pandemic would have a negative impact on the quality of their specialist education, and 14% had considered changing their profession due to the pandemic [8]. In India, 74.3% of trainees reported worry about meeting the goals of their specialty training due to reduction in surgical caseloads [7]. In Italy, anxiety about the professional future was expressed by 84% of the trainees, and 59% of them had the perception that their training was irreversibly compromised [1]. In the United Kingdom, 79% of trainees were concerned about the overall impact of COVID-19 on their training, with 56% expressing that their training progression may be adversely affected [10]. The study from the United States assessed the mental health of trainees and found that 80% reported a negative impact due to the pandemic, 44% experienced burnout, and 40% contemplated self-harm or knew a colleague who considered or attempted suicide [2].

The COVID-19 pandemic overwhelmed healthcare organisations to the point where essential personnel were being pulled from their primary professions to provide care to those affected by the virus. O&G PGTs were no exception. In a systematic review of the impact of COVID-19 on surgical training that included more than 20 countries with 5260 PGTs and 339 PDs, redeployment to non-surgical roles varied across studies from 6% to 35.1% [11]. Our review noted much higher rates, with the highest being in India [7]. Given the sudden and desperate need for providers, many of these PGTs were reassigned to COVID-19 care wards with little to no education or guidance. It was a common experience across countries for the PGTs to feel unprepared with inadequate training [5,9,10]. However, there were instances where despite being taken away from their primary duties as obstetricians, PGTs felt supported and prepared to treat COVID-19 patients [7]. Some trainees even felt that this reassignment allowed them to develop skills and competencies they otherwise may not have [3]. 

Countries handled relocation differently. In Brazil, the National Medical Residency Commission mandated that the relocation of clinical residents during the pandemic should be based on an incidence coefficient (number of new cases/million inhabitants) and the classification by the epidemiological complexity level of the municipality and health region of the medical residency programme [12]. This was because the situation of the COVID-19 pandemic differed between states and cities. In the United States, the Accreditation Council for Graduate Medical Education (ACGME) established three stages of operation for each sponsoring residency institution to allow them the flexibility to redeploy PGTs to areas of increased clinical need [13]. In the United Kingdom, over half of all O&G PGTs were redeployed to support frontline specialties such as Accident and Emergency and General Medicine [14]. It is also important to consider the potential positive impact that this role reassignment may have had on the overall education of the PGTs. The field itself is so uniquely specialised that PGTs do not often get the opportunity to address healthcare concerns that the general practitioner deals with on a day-to-day basis. The fact that the COVID-19 pandemic pushed many PGTs into intensive care or internal medicine units may lead to more well-rounded graduates. Though treatment for pregnant women often varies drastically from the standard-of-care set for non-pregnant adults, it may be useful for this generation of PGTs to have experienced a different side of critical care medicine that they would not have been exposed to. 

While O&G is largely a hands-on learning specialty, there is a role for formalised classroom learning. A major loss for many O&G programmes worldwide was their structured didactic curriculum. Several studies discussed not only the disruption to their established teaching methods but also the difficulties they faced in restructuring [5,8]. Despite reintroducing aspects of their educational programme in a virtual format, these courses were not offered in the same manner they were initially designed. This change caused a disruption in the educational flow for many residents, even resulting in longer residency periods for some [6]. Further, in some countries, PDs felt that they did not have access to a virtual supportive environment [5]. This limited their ability to disseminate courses even once they had been converted to a virtual format. In other countries, despite access to the internet, connectivity problems and increased distractions led to drawbacks to the new virtual curriculum [7]. Each country has its own standard for assessing PGTs' competency prior to graduation, and it may be useful to compare PGTs' academic performance prior to and after the pandemic. 

Another issue that appeared to be common throughout O&G programmes was that of a decrease in overall clinical training and exposure. With fear of the COVID-19 virus at an all-time high, many patients avoided attending clinic appointments unless absolutely necessary. Many hospitals limited surgical cases to only those that qualified as emergent. For many PGTs, the decreased number of surgical patients left them ill-prepared to confidently graduate from their training programmes [3,8]. In the United Kingdom, up to 33% of trainees felt the need to change their future career plans in response to decreased operating time and exposure to O&G [14]. Further, while some studies referenced the idea of utilising surgical simulators to fill the gap left by the decrease in actual surgery during the COVID-19 pandemic, there is little published data on how such training compares to hands-on surgery [7,9,10].

Positive takeaways from the COVID-19 pandemic 

Support and preparedness by both PDs and PGTs are key to preventing the disruption of postgraduate education. In the end, individual countries and their medical education bodies decide what is best. However, PDs can provide enhanced support through mentorship, flexibility in training schedules to reduce burnout, and revised training curriculums [4]. Lengthening of training programmes to account for time spent away from the specialty may be another solution [2]. 

Didactic education experienced many positive changes. Changing in-person didactic sessions to virtual education requires good technological support. Some countries provided swift, uncomplicated, good technical support and their experience needs sharing [6]. There were examples of excellent virtual curriculum with evening and weekend webinars inviting specialists from across the globe [15]. Many organisations provided weekly recorded educational sessions, not previously available to PGTs, and many engaged in sharing educational platforms or lectures with trainees across the nation [4]. Online video didactics and recorded lectures are extremely helpful allowing PGTs to watch at their own time and pace. Remote learning can decrease the stress associated with commuting to one location for education and then back to work elsewhere. Staff who previously might not have participated in trainee education could easily participate online [4].

Some countries were able to expand services to patients and thus offer continued training opportunities. For example, in the United Kingdom, the National Health Services (NHS) bought capacity from private hospitals, for non-urgent elective care that had been put on hold by NHS trusts swamped by patients with COVID-19 [16]. This helped some trainees to continue their hands-on skills. Loss of training opportunities in outpatient settings can be circumvented too. Tottenham Hotspur Football Club offered its stadium as a place to host maternity and antenatal services to allow hospitals to focus on COVID-19 and also allow PGTs to attend clinics [17].

The isolation impacts of COVID-19 also need to be acknowledged by PDs. Residency at baseline can be a lonely and mentally exhausting experience. ACGME has published a helpful book with considerations for promoting and maintaining the well-being of trainees. In a US study, 80% said mental health resources were available but only 29% used them [2]. Broader adoption of institutional resources should be encouraged. Resident-led wellness initiatives like providing discretionary time and promoting social events were the highest rated in supporting resident wellness [2]. Where possible, research projects should not be put on hold. Time spent at home can be considered as dedicated research time so future blocks could be changed to clinical time on returning to work [4].

The strengths of this study included a diverse global sampling of the effects of the pandemic on PGT in O&G. We included the voices of trainees and PDs. There are inherent limitations to the study. The quality of the survey studied included in the paper is unfortunately poor, as all 10 studies used unvalidated surveys. The response rates of most of the studies either were not reported or were poor (<60%), though some studies had a 69-73% response rate. This makes it difficult to draw conclusions about these studies. Moving forward, it would be interesting to conduct survey-based data from countries missing in this evaluation to get a broader picture of how COVID-19 has changed the postgraduate experience for trainees in different locations.

## Conclusions

The COVID-19 pandemic led to the restructuring of O&G programmes worldwide. Most surveys were conducted during the pandemic. Our review discusses O&G education, but we have also looked at papers from other surgical specialties without including them. Trainees in all nations reported deployment to support the front lines and faced a decrease in educational sessions, clinical training, and surgical caseload. These changes had negative repercussions, increasing anxiety and stress, but there were positive lessons too. Only time will tell if the pandemic affected O&G education adversely. Further follow-up on the cohort of PGTs now in practice may lend useful information on the long-term implications. Future research could look at the effect of the pandemic on scores on board examinations, and self-reported comfort levels and patient outcome details of these graduating fellows would be useful. 
